# CheapStat: An Open-Source, “Do-It-Yourself” Potentiostat for Analytical and Educational Applications

**DOI:** 10.1371/journal.pone.0023783

**Published:** 2011-09-13

**Authors:** Aaron A. Rowe, Andrew J. Bonham, Ryan J. White, Michael P. Zimmer, Ramsin J. Yadgar, Tony M. Hobza, Jim W. Honea, Ilan Ben-Yaacov, Kevin W. Plaxco

**Affiliations:** 1 Department of Chemistry and Biochemistry, University of California Santa Barbara, Santa Barbara, California, United States of America; 2 Department of Electrical and Computer Engineering, University of California Santa Barbara, Santa Barbara, California, United States of America; 3 Program in Biomolecular Science and Engineering, University of California Santa Barbara, Santa Barbara, California, United States of America; University of Pennsylvania, United States of America

## Abstract

Although potentiostats are the foundation of modern electrochemical research, they have seen relatively little application in resource poor settings, such as undergraduate laboratory courses and the developing world. One reason for the low penetration of potentiostats is their cost, as even the least expensive commercially available laboratory potentiostats sell for more than one thousand dollars. An inexpensive electrochemical workstation could thus prove useful in educational labs, and increase access to electrochemistry-based analytical techniques for food, drug and environmental monitoring. With these motivations in mind, we describe here the CheapStat, an inexpensive (<$80), open-source (software and hardware), hand-held potentiostat that can be constructed by anyone who is proficient at assembling circuits. This device supports a number of potential waveforms necessary to perform cyclic, square wave, linear sweep and anodic stripping voltammetry. As we demonstrate, it is suitable for a wide range of applications ranging from food- and drug-quality testing to environmental monitoring, rapid DNA detection, and educational exercises. The device's schematics, parts lists, circuit board layout files, sample experiments, and detailed assembly instructions are available in the supporting information and are released under an open hardware license.

## Introduction

Potentiostats, the cornerstone of electrochemistry research, have seen little adoption in resource poor environments such as the developing world and teaching laboratories. An important factor in this is cost, with academic labs and industrial groups typically paying upwards of US $10,000 for a general-purpose, research quality workstation. Indeed, even the least expensive commercially available laboratory potentiostats (*e.g.*, Dagan Chem-Clamp, DropSens μSTAT 200) sell for more than a thousand dollars and achieve only limited functionality. An inexpensive instrument that is versatile enough to generate standard waveforms could thus significantly broaden the use of electrochemistry in resource-poor environments. Such a device would enable, for example, quantitative measurements in food and drug quality control [1], the analysis of trace metals [2], environmental monitoring [3], and the construction of simple biosensors [4] [5] [6] [7] [8].

While a do-it-yourself device cannot rival the quality and capabilities of research-grade potentiostats, many of the features of top-of-the-line commercial instruments are unnecessary for environmental or public health applications. Supporting this argument, simple potentiostats such as the home glucose meter can make reasonably precise electrochemical measurements at a cost of less than US $100. The software and hardware designs underlying these devices, however, are proprietary and thus are not easily modified to support other applications. Given these arguments, it is perhaps not surprising that a number of open-source potentiostats have been described in the scientific literature over the last four decades [9] [10] [11] [12]. For example, in 1980 Bond and Norris published a description of a waveform generator that employed inexpensive integrated circuits [13], followed by Brown who, in 1982, described a more sophisticated computer-controlled instrument [14]. More recent open-source potentiostat development has focused largely on miniature potentiostats, often with implantation in mind [15] [16] [17] [18] [19] [20]. These devices, however, typically do not offer a wide variety of waveforms, nor do they employ modern computer interfaces. Other recently described, open-source potentiostats employ custom-made microelectronics, rendering them ill suited for use in resource limited-applications [21] [22] [23]. Thus, despite significant literature precedent there remains an unmet need for an inexpensive, easily built potentiostat supporting the commonly employed electrochemical waveforms. Indeed, the desire for a low-cost, open-source, general-use potentiostat is highlighted by a recent academic attempt to “re-purpose” off-the-shelf glucose meters to support new analytical applications [24].

**Figure 1:**
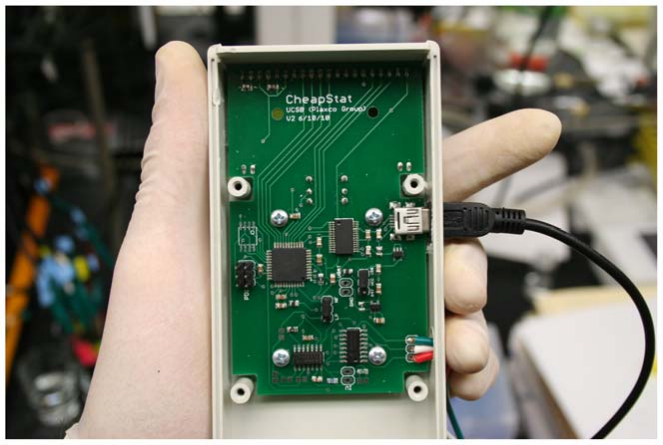
The CheapStat, an inexpensive, “do-it-yourself” potentiostat that can be built for under $80. The device supports cyclic, square wave, linear sweep and stripping voltammetry over the potential range −990to +990 mV and over frequencies from 1 to 1000 Hz. The device supports a range of environmental, food and drug quality control, and educational applications. The reverse of the circuit board supports a three-line LCD display and a joystick, which is used to select an experiment protocol, change its parameters (frequency, starting voltage, end voltage, scan rate), and initiate the experiment.

In response to the apparent need for a truly inexpensive, fully programmable potentiostat we present here the CheapStat (**Figure 1**), an open-source potentiostat easily constructed by anyone proficient at assembling circuits. We believe this device may prove of value in undergraduate chemistry labs, the developing world and other environments where resources are limited. In support of this, we have demonstrated the device's utility in applications including food and drug testing, environmental monitoring, education, and biosensing.

## Materials and Methods

With the exception of the printed circuit boards, which were custom ordered from PCBex (Houston, TX ∼30 each), all of the components required to build the CheapStat were obtained from Mouser Electronics (Mansfield, TX) at a total cost of ∼$80 per device in small numbers. The schematics for this device (Layout S1, Layout S2, Layout S3, Layout S4, Layout S5, Layout S6, Layout S7), and instructions for building it (Appendix S1), are available in the supporting information and released under an open hardware license. The necessary software (runtime and source code), and other updates, can be found on our website (http://www.chem.ucsb.edu/~kwp/cheapstat/).

### Measurements of Ascorbic Acid in Orange Juice

As a simple demonstration of the educational applications of the CheapStat, we have used it to measure the ascorbate concentration in orange juice. Because ascorbic acid is redox active its level in orange juice can be measured via cyclic voltammetry [1] using the method of standard additions. Doing so represents a safe, simple experiment suitable for a general chemistry or even high school lab course.

In this experiment, four orange juice samples were prepared, one of which was unmodified and the remaining three of which were modified via the addition of exogenous ascorbic acid at 0.1, 0.2 or 0.3 M. To increase their conductivity potassium chloride was added to each of these to 1 M. A graphite pencil “lead” taken from a mechanical pencil was used as the working electrode. A standard Ag/AgCl reference and platinum counter electrode were used. Measurements were made in 5 mL of the various orange juice samples (Minute Maid Original, Atlanta, Ga). Cyclic voltammetry measurements were taken from 200 to 900 mV, and the current at 550 mV was used for quantification of the orange juice. Of note, although it is the most abundant redox active species in orange juice, ascorbate is not the only redox active compound present. Specifically, other substances interfere with these measurements at potentials above 600 mV and thus ascorbate should not be determined using readings collected above 600 mV.

**Figure 2:**
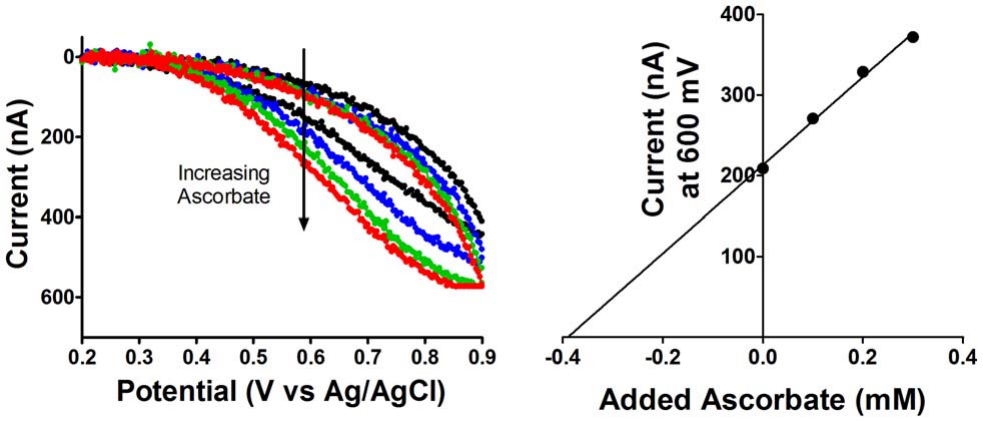
The CheapStat supports cyclic voltammetry. Shown is the CV-based measurement of ascorbic acid (Vitamin C) in orange juice, using the method of standard additions. (Left) To do so, 0, 5, 10, and 15 mL of an ascorbic acid standard solution (at 0, 0.1, 0.2, and 0.3 M in orange juice) were added to a 25 mL sample of name-brand orange juice and interrogated via cyclic voltammetry performed using an inexpensive pencil “lead” as the working electrode. (Right) A linear extrapolation of the oxidation current observed at 600 mV was used to determine the initial ascorbate concentration, which at 1.95±0.11 mM is quite close to the “60 mg per serving” (2 mM) indicated on the manufacturer website.

To determine the initial ascorbate we make a linear plot of current at 550 mV versus added ascorbate (**Figure 2**). Extrapolation of this line to 0.39 mM for the diluted sample indicates that our orange juice sample contained 1.95±0.11 mM of ascorbic acid, a convincing figure given the concentration (2 mM) listed on the manufacturer website.

### Monitoring Redox of Ferricyanide Using Cyclic Voltammetry

The voltammetric response and redox cycling of the reversible ferricyanide-ferrocyanide couple is a commonly used educational tool to introduce electrochemistry, and here we demonstrate this reaction using the CheapStat. In addition to its pedagogical roles, ferricyanide is a common oxidant in organic chemistry and has been used as a probe for a variety of organic and biological reactions, as the reduction of ferricyanide to ferrocyanide is easily monitored.

In this experiment, we dissolved potassium ferricyanide in 50 mL of 1 M potassium nitrate to final concentrations of 1 and 2 mM. As our working electrode we employed a simple platinum disk electrode. To further highlight the low cost and self-assembled nature of the CheapStat, these experiments were performed independently with either a standard, commercial Ag/AgCl electrode or a hand-fabricated glass pipette-based Ag/AgCl electrode (prepared as in [25]). A length of platinum wire was employed as the counter electrode, although we note that many conductive materials would serve equally well as simple counter electrodes. Using these electrodes we conducted cyclic voltammetry over the potential range from 0 to 650 mV at a rate of 10 mV/sec according to the parameters established by Bott and Jackson [26].

**Figure 3:**
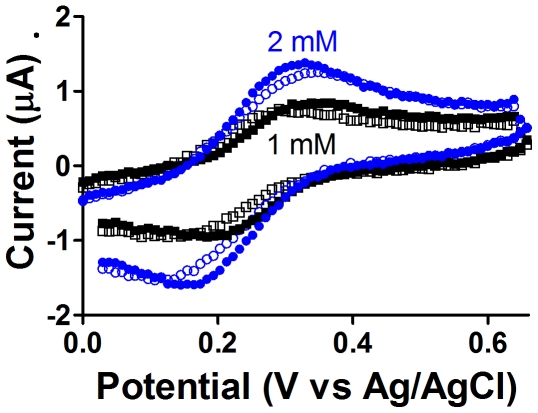
The CheapStat is compatible with common educational experiments. Shown is the cyclic voltammetry-based monitoring of potassium ferricyanide in 1 M potassium nitrate solution, using both commercial (filled symbols) and hand fabricated (open symbols) Ag/AgCl reference electrodes. Ferricyanide concentrations of 1 mM (black squares) and 2 mM (blue circles) were interrogated via cyclic voltammetry and show the characteristic voltammetric response of a fully reversible reaction.

The ferricyanide redox response forms the characteristic “duck” shape of a fully reversible reaction in the cyclic voltammogram (**Figure 3**). Both the commercially available Ag/AgCl reference and a homemade reference demonstrate results in close agreement when observing the ferricyanide reaction at the tested concentrations.

### Analysis of Acetaminophen Content in Over-the-Counter Pain Medication Using Linear Sweep Voltammetry

As a final demonstration of the CheapStat's educational applications, we have used it to measure the acetaminophen content of a painkiller tablet. Acetaminophen is a redox active [27] anti-inflammatory medication that can be quantified by linear sweep voltammetry. Here we analyzed the acetaminophen content of an over-the-counter pain reliever using the method of standard additions.

In this experiment we dissolved a commercial pain reliever (stated to contain 500 mg acetaminophen) in 250 mL of 2 M sulfuric acid to a final theoretical concentration of 13.33 mM. Splitting this into four samples we adulterated three with increasing amounts of exogenous, pure acetaminophen at 0.002, 0.005 and 0.01 M. As our working electrode we employed a Teflon-coated gold wire of diameter 0.08 mm, a 1 cm length of which was heated to exposed the bare gold surface. A standard Ag/AgCl electrode and a length of platinum wire were employed as the reference and counter electrode respectively. We conducted linear sweep voltammetry on these samples over the potential range from 500 to 1000 mV at a rate of 10 mV/sec.

**Figure 4:**
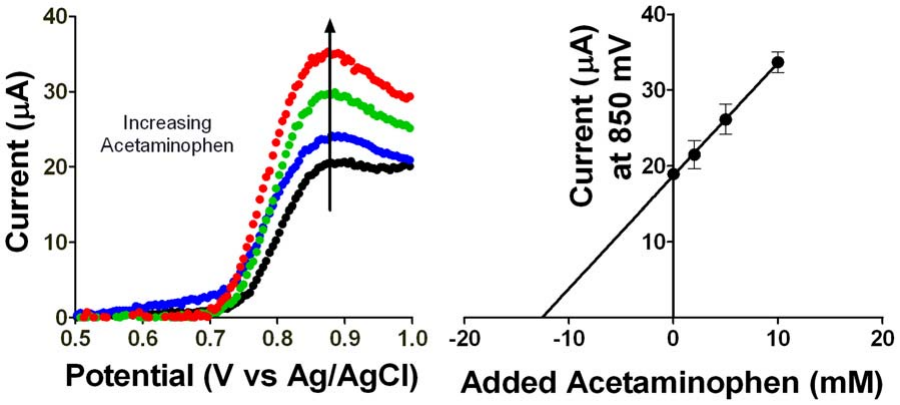
Analysis of the acetaminophen content of an over-the-counter painkiller using linear sweep voltammetry. An acetaminophen tablet was crushed and dissolved in sulfuric acid. Standards were added to three solutions, and one was left untouched. (Left) Linear sweep voltammetry performed with a gold-wire working electrode was used to measure the acetaminophen concentration by the method of standard additions. (Right) A linear extrapolation of the oxidation current observed at 850 mV was used to determine the initial acetaminophen concentration in a 250 mL solution containing one crushed tablet, which at 12.6±1.7 mM (472±63 mg per tablet) is close to the 500 mg per tablet reported by the manufacturer.

To calculate the acetaminophen concentration in our unadulterated sample we linearly extrapolate the current observed at 850 mV (**Figure 4**). Extrapolation and conversion to total quantity shows that each tablet contained 472±63 mg of acetaminophen, which is in close agreement with the amount of acetaminophen reported on the label of the commercial product we employed.

### Measurement of Arsenic Using Anodic Stripping Square Wave Voltammetry

In some rural parts of India and Bangladesh, arsenic in drinking water is a major health concern. We have used the CheapStat to measure arsenic [2] levels in lake water. In all areas of the world, the recommended maximum level of arsenic in drinking water is 10 ppb.

Samples of lake water were collected from Lake Cachuma (Santa Barbara, CA) and stored in Nalgene polyethylene bottles. The lake water was acidified by mixing it with concentrated hydrochloric acid in an 11 to 1 ratio. Gold disk electrodes (CH Instruments, Austin, TX) were polished in a slurry of 0.5 micron alumina on a polishing cloth and then rinsed thoroughly with deionized water. A sealed ampoule of arsenic oxide (Fluka Product #38150, via Sigma Aldrich, St Louis, Mo) was diluted 1000 fold with acidified lake water. Aliquots of the diluted standard were added to 5 mL samples of acidified lake water. The concentration of added arsenic in the samples was 5 ppb and 20 ppb.

**Figure 5:**
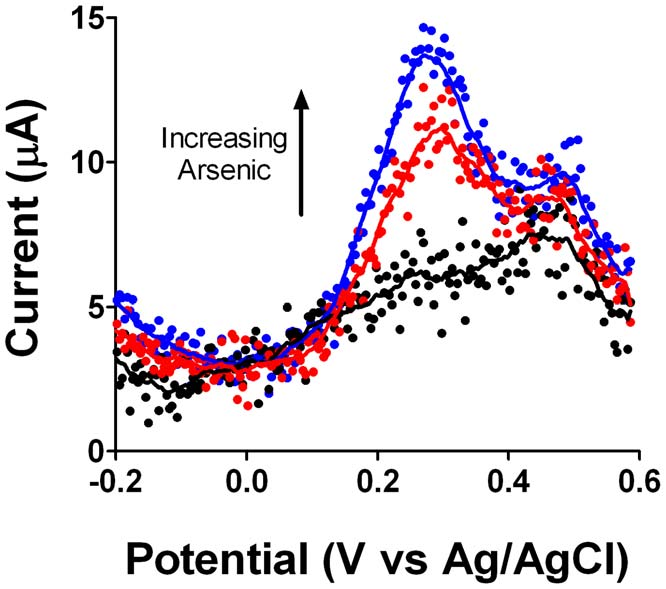
Analysis of the arsenic content in three acidified lake water solutions. Samples were collected from Lake Cachuma (Santa Barbara, CA) and mixed in an 11∶1 ratio with concentrated HCl. An arsenate standard was spiked into two of the samples (top 20 ppb, middle 5 ppb), and one was left as a blank (bottom). The dissolved arsenate in each solution was reduced at −0.5 V and then analyzed by square wave voltammetry at 40 Hz.

In each lake water sample, a gold disk, platinum counter, and silver/silver chloride reference electrode were immersed. The current at the working electrode was held at −0.500 mV for 120 seconds while the solution was mixed by pipetting. And then a square wave measurement at 40 Hz was run from −270 mV to 600 mV. The CheapStat was easily able to make measurements at spiked 5 and 20 ppb arsenic concentrations (**Figure 5**). There was no detectable arsenic in the lake water samples.

### Construction of a Simple E-DNA Biosensor and its Interrogation Using Square Wave Voltammetry

Electrochemical DNA (E-DNA) biosensors are inexpensive, reusable DNA detectors [4]. They can make quantitative measurements in complex mixtures such as blood, urine, saliva, and PCR reaction products. In this experiment, we have used an E-DNA sensor to detect a synthetic DNA oligonucleotide with a sequence found within the *salmonella* genome.

**Figure 6:**
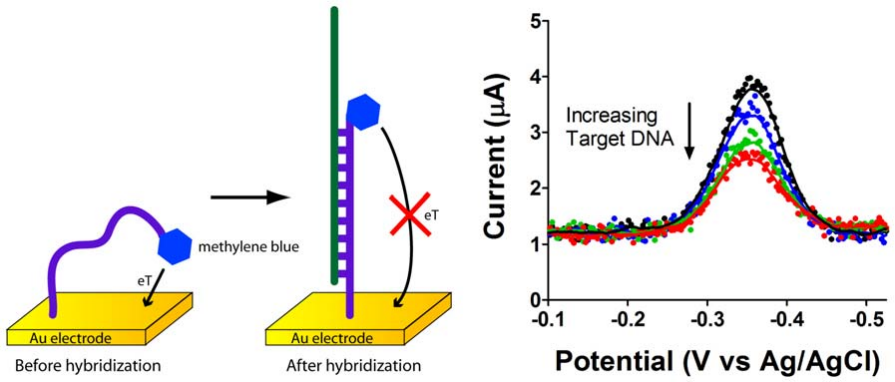
The electrochemical detection of a specific DNA sequence directly in PCR mix. An E-DNA sensor (left) was interrogated via square wave voltammetry. Scanning the E-DNA biosensor at 100 Hz before and after the addition of complimentary target (20, 60, and 200 nM in PCR mix) clearly indicates the presence of the target DNA: over the course of an hour, hybridization with the analyte (at 200 nM) caused a 61% decrease in the peak current.

The sensing element in these devices is a synthetic 17-base oligonucleotide commercially synthesized with a thiol on its 5′-terminus and a methylene blue reporter on the opposite terminus. The thiol readily anchors the oligonucleotide to a gold electrode (via very facile self assembled monolayer chemistry), leaving the other end free to approach the electrode and transfer electrons (**Figure 6**). If a target molecule hybridizes with this system, the efficiency with which the methylene blue approaches–and thus transfers electrons to- the electrode surface decreases, decreasing the observed rate of electron transfer [28]. As square wave voltammetry is very sensitive to changes in electron transfer kinetics, it is ideally suited to monitor biosensors in this class [5].

The DNA probe employed in this sensor was synthesized by Biosearch Technologies (Novato, CA), and the target by Sigma Genosys, a division of Sigma Aldrich (St Louis, MO). The sequences of the oligonucleotides were:

LinearSensor 5′-HS−(CH_2_)_6_−TGGATCGGCGTTTTATT−(CH_2_)_7_−NH−MB-3′ and
TargetDNA 5′-TTGAATATCTGAACAAGAATAAAACGCCGATCCA-3′.

To fabricate the sensor we polished gold disk electrodes using 0.05 µm alumina before cleaning them electrochemically in 0.5 M H_2_SO_4_ and 0.5 M H_2_SO_4_ with 0.01 M KCl with a series of cyclic voltammetry scans. Each electrode was then rinsed in deionized water before immersion in a 200 nM solution of the relevant DNA probe in pH 7.4 phosphate buffered saline. After an hour the electrodes were rinsed with deionized water and then stored in 2 mM mercaptohexanol (to complete the formation of the self-assembled monolayer) overnight. Before use the sensors were rinsed in deionized water.

The sensors were immersed in 5 mL of phosphate buffered saline. An initial square wave voltammetry scan was made from 0 to −0.5 V at 100 Hz. We then challenged the sensor by adding 100 µL of a 10 µM stock of a 34-base analyte DNA. At 30 min and 60 min after mixing we recorded new square wave scans, observing the expected 60% decrease in signal after 1 hour. These results were in good agreement with our earlier work, which used a CH Instruments 630B (Austin, Texas) potentiostat [5].

## Results and Discussion

The heart of the CheapStat is a closed-loop analog control circuit, capable of regulating electrode voltage with sub-millivolt precision as it measures electrode current with nanoamp precision. This control circuit is driven by an Atmel XMEGA microcontroller containing a Digital to Analog Converter and an Analog to Digital Converter of sufficient precision to support the relevant voltage waveform generation and current quantification. Coupled to this microcontroller a Universal Asynchronous Receiver-Transmitter to Universal Serial Bus (USB) chip provides a convenient interface between this microcontroller and a data analysis computer via a USB port. An operational amplifier feedback system sets the voltage across the electrochemical cell and supplies the current needed to drive the electrochemical reaction. We employed Texas Instruments TLC2262CP operational amplifiers (Mouser Electronics) in the CheapStat because they are low power and require little input current. Specifically, these amplifiers have an input bias current of only 1pA, which permits reliable sensing of low nanoamp currents.

Inherent in the specific device choices described above is the overarching design theme of the CheapStat: ease of fabrication, modification and use. For example, using the above-described components and an inexpensive, custom-built (and easily obtained) PC board we have hand fabricated a number of CheapStats without the use of a reflow oven, wave soldering, or other sophisticated circuit assembly tools. The CheapStat firmware is likewise easily updated with the aid of a simple microcontroller programming kit. Finally, the designs of the CheapStat are available under a Creative Commons license, and the attached documentation is detailed enough to support efforts to build the CheapStat or expand upon its design.

As currently implemented the CheapStat supports square wave, linear sweep, stripping, and cyclic voltammetry over potentials from −990 mV to 990 mV, frequencies from 1 to 1000 Hz, and currents from ∼100 nA to 50 µA. These operation ranges can be expanded with simple hardware adjustments. In the event that a given experiment produces currents greater than 50 µA, for example, a simple resistor can be added in series with the working electrode to bring the current back into the useful range of the device. The CheapStat is easy to use: it includes a three-line LCD display and a joystick by which the operator selects the specific protocol, defines its parameters (frequency, starting voltage, end voltage, scan rate), and initiates the experiment. Copying data from the CheapStat onto a computer is similarly easy, requiring only a single mouse click. Finally, the CheapStat is hand-held, weighs only 115 grams, is powered via its USB port, and it can be controlled by a simple laptop or netbook computer, rendering the device portable and field ready.

To demonstrate the utility of the CheapStat we have conducted a set of simple experiments spanning a range of representative applications. These include the use of cyclic voltammetry and an inexpensive, pencil “lead” working electrode to measure vitamin C (ascorbic acid) in orange juice (**Figure 2**), cyclic voltammetry to observe the reversible ferricyanide/ferrocyanide reaction (**Figure 3**), and linear sweep voltammetry to determine the acetaminophen (paracetamol) content of an over-the-counter painkiller (**Figure 4**) –experiments typical of those found in undergraduate laboratory courses. To illustrate the ability of the CheapStat to support more complex analytical applications, we have also demonstrated the use of anodic stripping square wave voltammetry to analyze the arsenic content in several spiked lake water solutions (**Figure 5**) and square wave voltammetry to measure the concentration of a specific DNA sequence directly in PCR mix using a simple electrochemical DNA (E-DNA) biosensor (**Figure 6**).

The CheapStat may prove of particular value as an educational tool. In each of the above experiments, for example, we obtained results of sufficient quality and reproducibility for facile interpretation by people who have little experience with chemical benchwork. Moreover, from an educational standpoint, the difficulty of using the CheapStat is quite low, rendering it quite suitable for college freshmen, or even high school students. With the increasingly significant role that electrochemistry plays in alternative energy and analytical technologies, the importance of accessible access to electrochemistry continues to grow. Electrochemistry likewise provides a powerful vehicle for teaching students about a broad range of topics within chemistry, including thermodynamics, redox reactions, reaction kinetics and titrations. Given these observations, we believe the availability of an inexpensive potentiostat such as that described here could have a positive impact on chemical education.

Beyond educational use, the CheapStat may also prove useful for analytical applications in the developing world. As we have shown, for example, the CheapStat supports the determination of arsenic in lake water^2^, an all too common environmental contaminant across broad areas of South Asia, where groundwater contamination is a pressing problem [29] [30] [31]. Indeed, while the development of low-cost approaches to remove arsenic from drinking water are well established, quantitative assays to determine the effectiveness of such treatment are cumbersome, requiring bulky instruments that cannot be brought into the field [32]. The CheapStat can analyze arsenic in minutes at concentrations well below the 10 ppb limit set by the US Environmental Protection Agency and World Health Organization.

### Conclusions

The CheapStat represents one example of a growing trend towards low-cost, easily fabricated analytical devices. Recent years have seen, for example, the development of inexpensive microfluidic devices made from polystyrene sheets [32] [33] [34] [35], paper [24] [36] [37] [38] [39] [40] and even gelatin [41]. Agrawal and Ugaz [42] [43] [44] have even developed an extremely inexpensive and portable PCR machine, which replaces an expensive Peltier chip with a ring shaped reaction vessel and three heating zones set at fixed temperatures. Finally, inexpensive surface enhanced Raman spectroscopy substrates [45] [46] centrifugation equipment [47], and spectrometers [48] [49] have all been reported in the recent literature. Moving forward, it is easy to imagine that technologies such as these could be integrated with the CheapStat to increase their utility in advanced analytical applications, including medical diagnostics.

## Supporting Information

Appendix S1 **Instructions for building a CheapStat, parts list, circuit diagrams and user notes.**
(DOCX)

Layout S1 **Schematics that can be used to view or order the necessary printed circuit board.**
(GBL)

Layout S2 **Schematics that can be used to view or order the necessary printed circuit board.**
(GBS)

Layout S3 **Schematics that can be used to view or order the necessary printed circuit board.**
(GTL)

Layout S4 **Schematics that can be used to view or order the necessary printed circuit board.**
(GTO)

Layout S5 **Schematics that can be used to view or order the necessary printed circuit board.**
(GTS)

Layout S6 **Schematics that can be used to view or order the necessary printed circuit board.**
(OLN)

Layout S7 **Schematics that can be used to view or order the necessary printed circuit board.**
(TXT)
