# Supplementary material for: CheapStat: An Open-Source, “Do-It-Yourself” Potentiostat for Analytical and Educational Applications
Source: PLoS One. 2011 Sep 13;6(9):e23783. doi: 10.1371/journal.pone.0023783 (PMC3172209; doi:10.1371/journal.pone.0023783)
Supplement: Appendix S1 — Instructions for building a CheapStat, parts list, circuit diagrams and user notes. (DOCX) [file pone.0023783.s001.docx]

General Instructions for building a CheapStat portable potentiostat.

**1// Circuit Board**

Send the Gerber files to a printed circuit board manufacturer, and order one or more of the CheapStat printed circuit boards. The gerber files are available as supporting information, and updated versions may be found on our website.

**2// Parts List**

Order the parts from a vendor such as digikey or Mouser electronics. See table S1 for a full list of parts. Some of these components may be substituted or omitted. For instance, this device can function with a wide variety of different flash chips.

**3// Soldering**

Solder all of the components onto the printed circuit board as indicated in figure S1. You can zoom in on this schematic, or look at the gerber files and the photos below (S2 and S3) for further clarification. If you do not have the appropraite soldering equipment, contract circuit assembly firms such as Sandberg Industries (Santa Ana, California) can do it for you for a reasonable fee. Be sure to wear an antistatic wrist strap when handling the components.

**4// Mounting the board.**

You will need to cut several holes in the enclosure (Fig S2 and S7) to handle the LCD display, USB port, and control switch. This can be done with a dremmel rotary tool.

**5// Programming the microcontroller.**

On the circuit board, there are a set of six pins that can be plugged into a microcontroller programmer. First, attach the cheapstat to a USB power source. Then, attach the microcontroller programmer. Finally, upload the firmware files to the microcontroller (available in supporting information). This process should take a few minutes. This is the final step of the assembly process.

**6// Using the cheapstat**

With the CheapStat plugged into the USB port of a computer, start the control software program. Use the four way switch on the CheapStat to select an experiment mode such as Square Wave Voltammetry, change the parameters, and run an experiment. Be sure that the control software is set to the correct COM port.

**7// Handling Data**

Data from the cheapstat can be copied directly into Microsoft Excel, Open Office, or other spreadsheet programs.


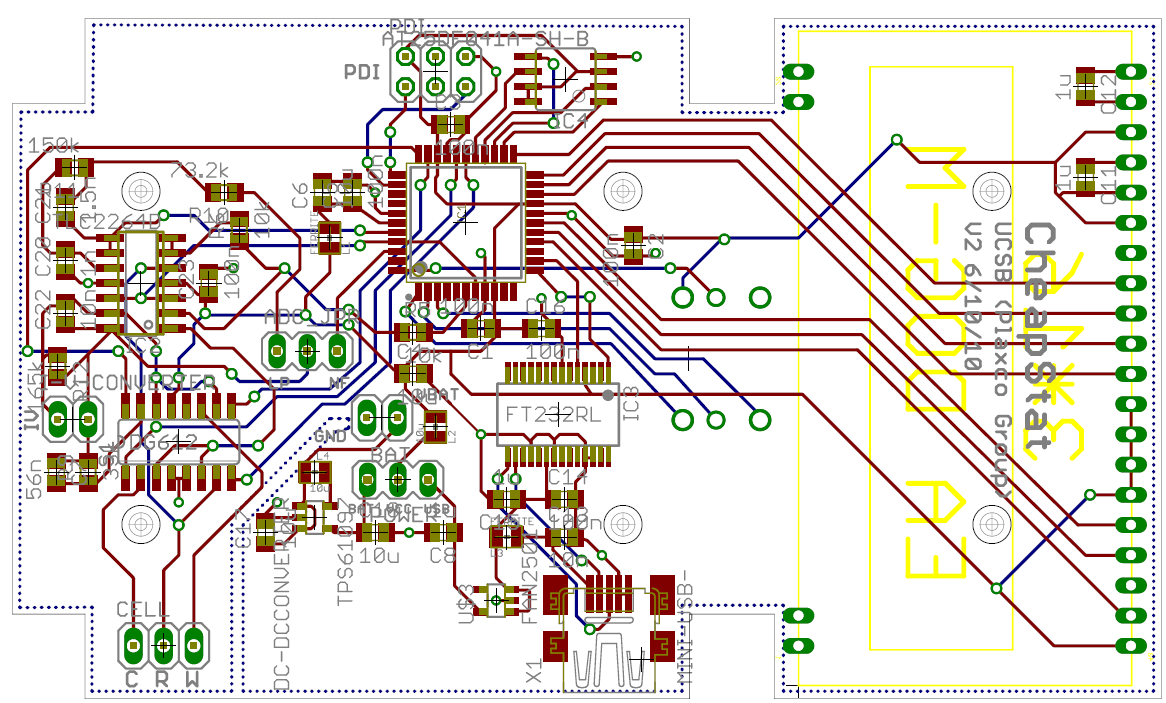


Figure S1 – Board layout. An Atmel XMEGA microcontroller containing a DAC and an ADC with precision sufficient for voltage waveform generation and current quantification. An RS-232 chip provides an interface between this microcontroller and a data analysis computer via a USB port. An op amp feedback system sets the voltage across the electrochemical cell and supplies the voltage needed to drive the electrochemical reaction.


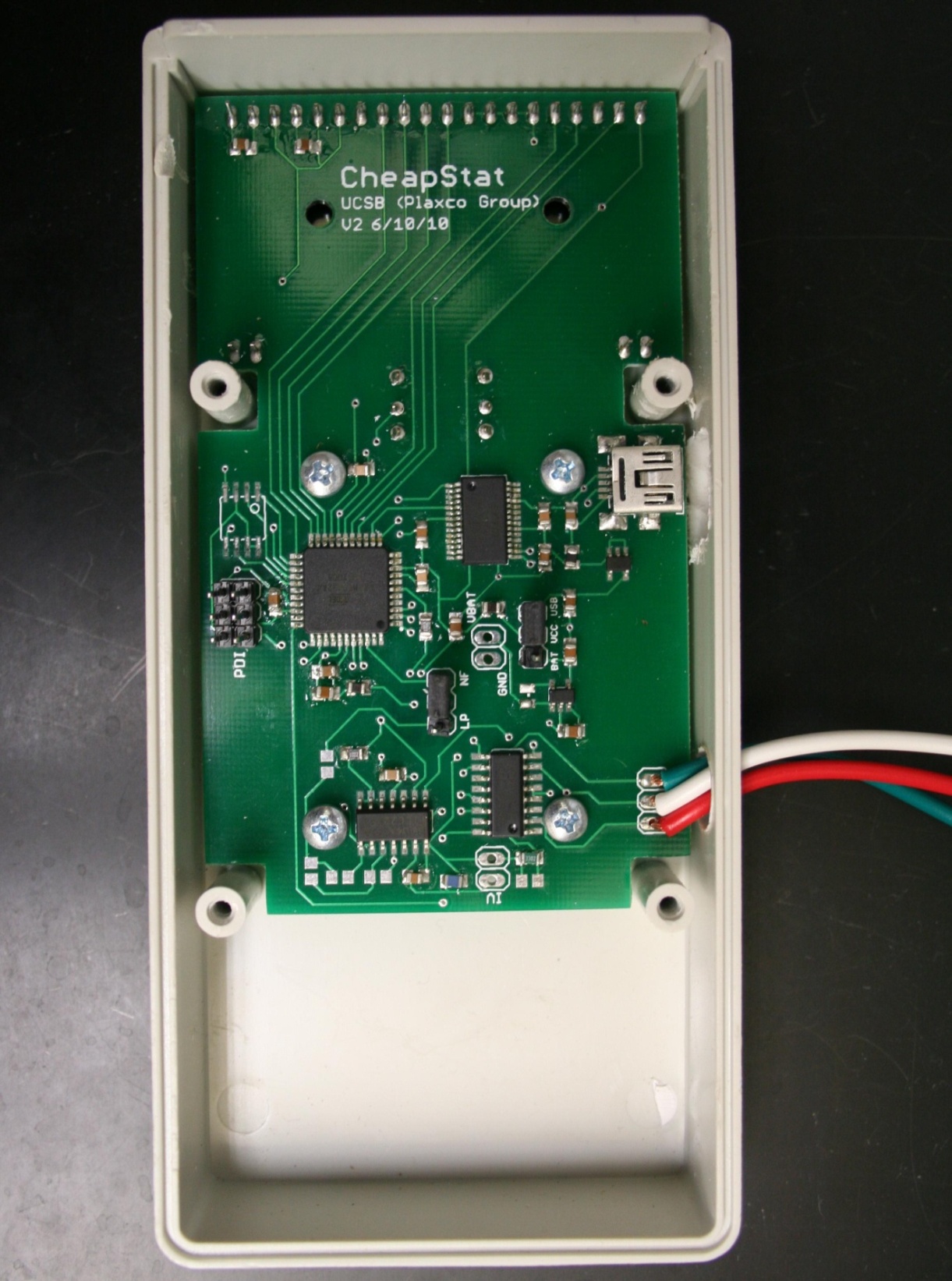


Figure S2 – The front side of the circuit board has a mix of surface mount and through-hole solder connections. Many of the components have a fine pitch, meaning that the electrical contacts are very close together. Nevertheless, all of the components were placed by hand and soldered without the assistance of a reflow oven.


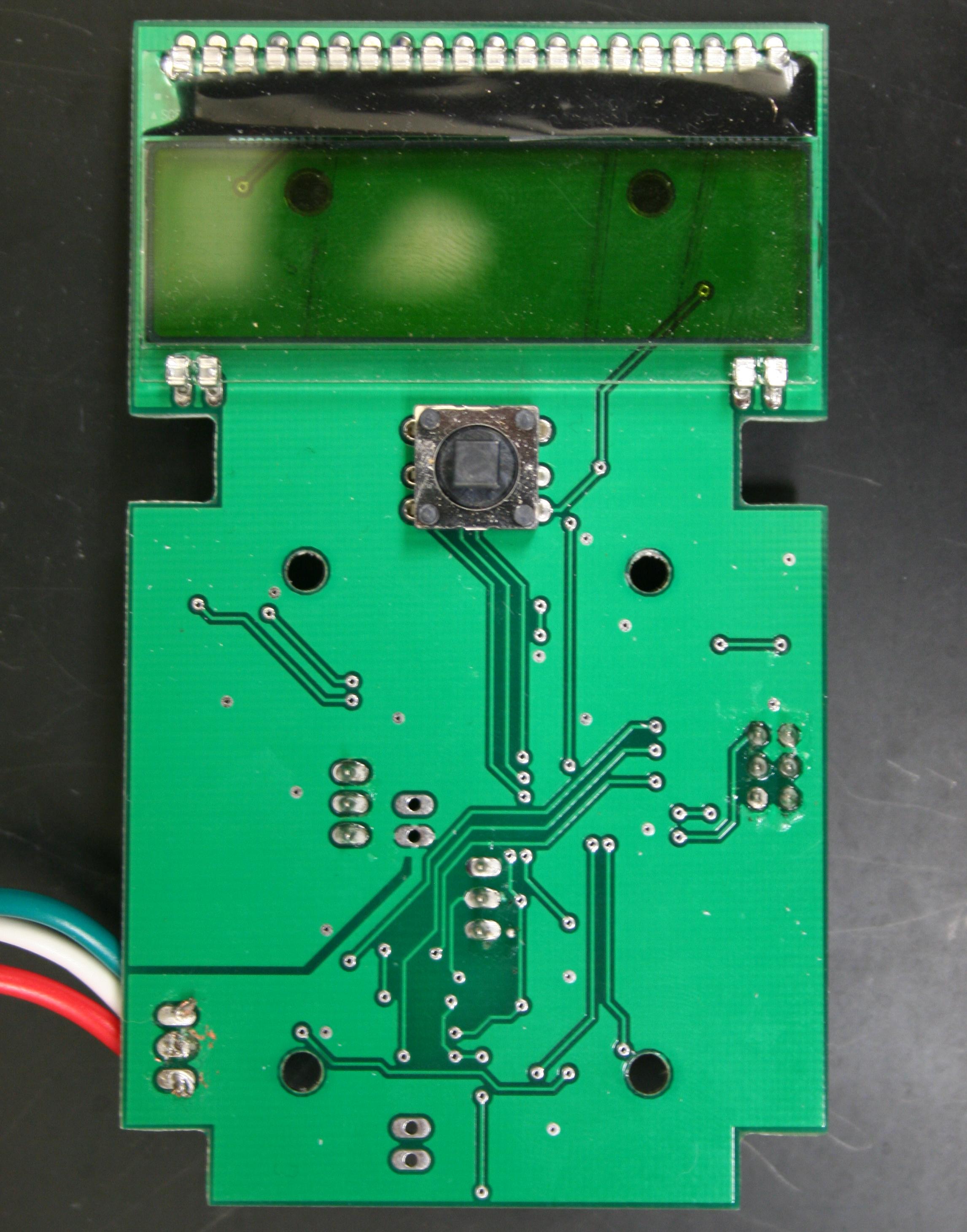


Figure S3 – The reverse of the circuit board supports a three-line LCD display and a joystick, which is used to select an experiment protocol, change its parameters, and initiate experiments.


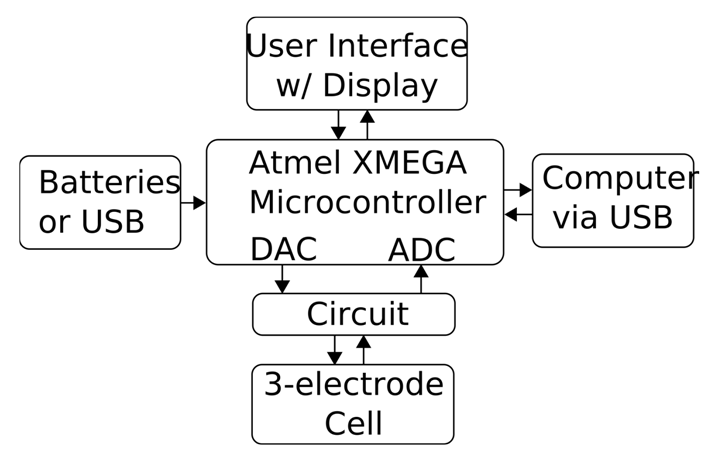


Figure S4 – A block diagram shows the relationship between the microcontroller and other components.


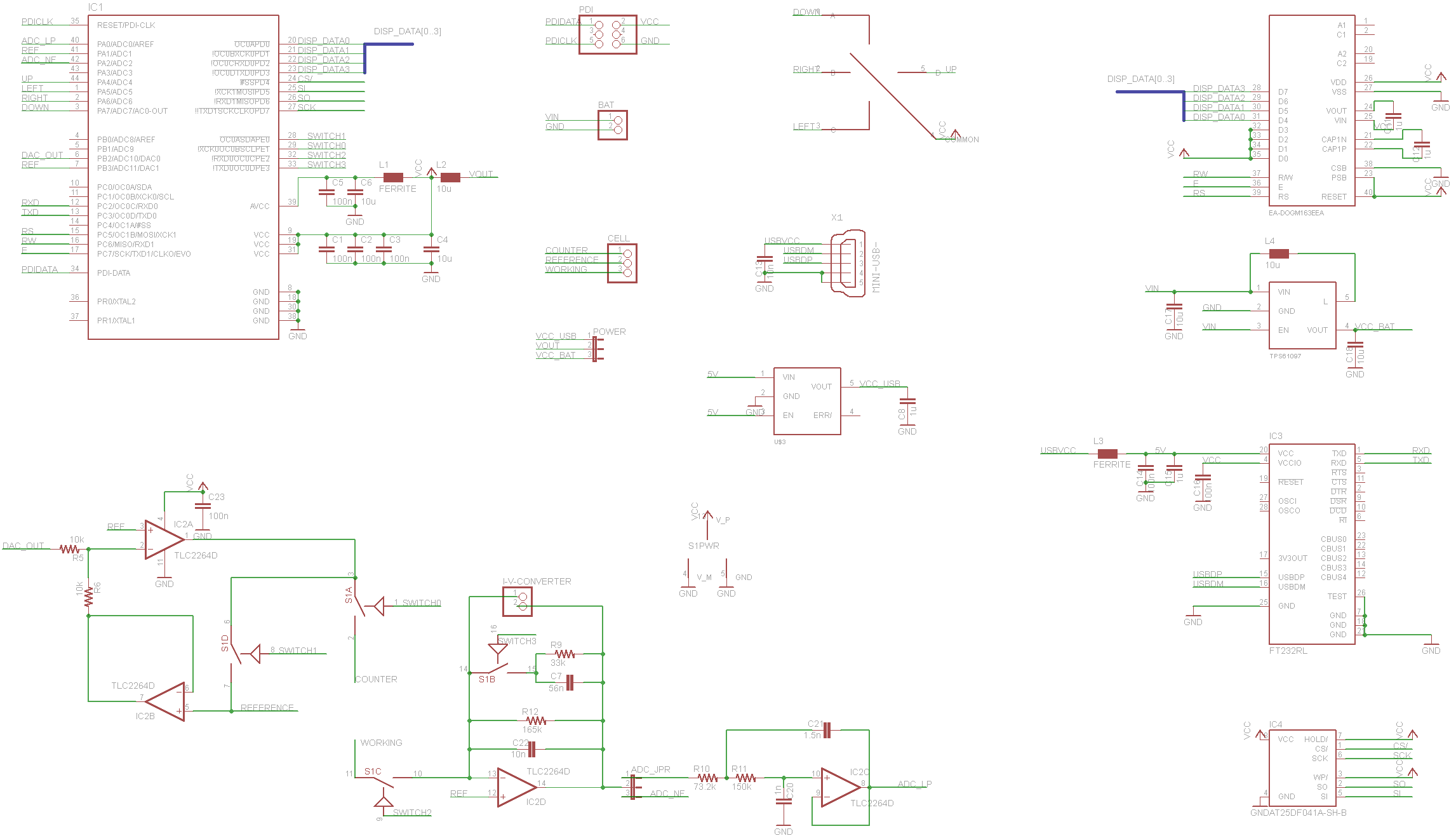


Figure S5 – A circuit schematic, detailing the connections between each of the main components. Zoom in to see the details.


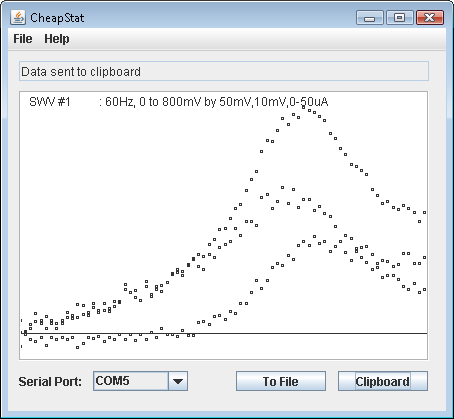


Figure S6 – Screen shot of the control software in action. Data can be copied to the computer clipboard with a single mouse click or saved into a new file within seconds.


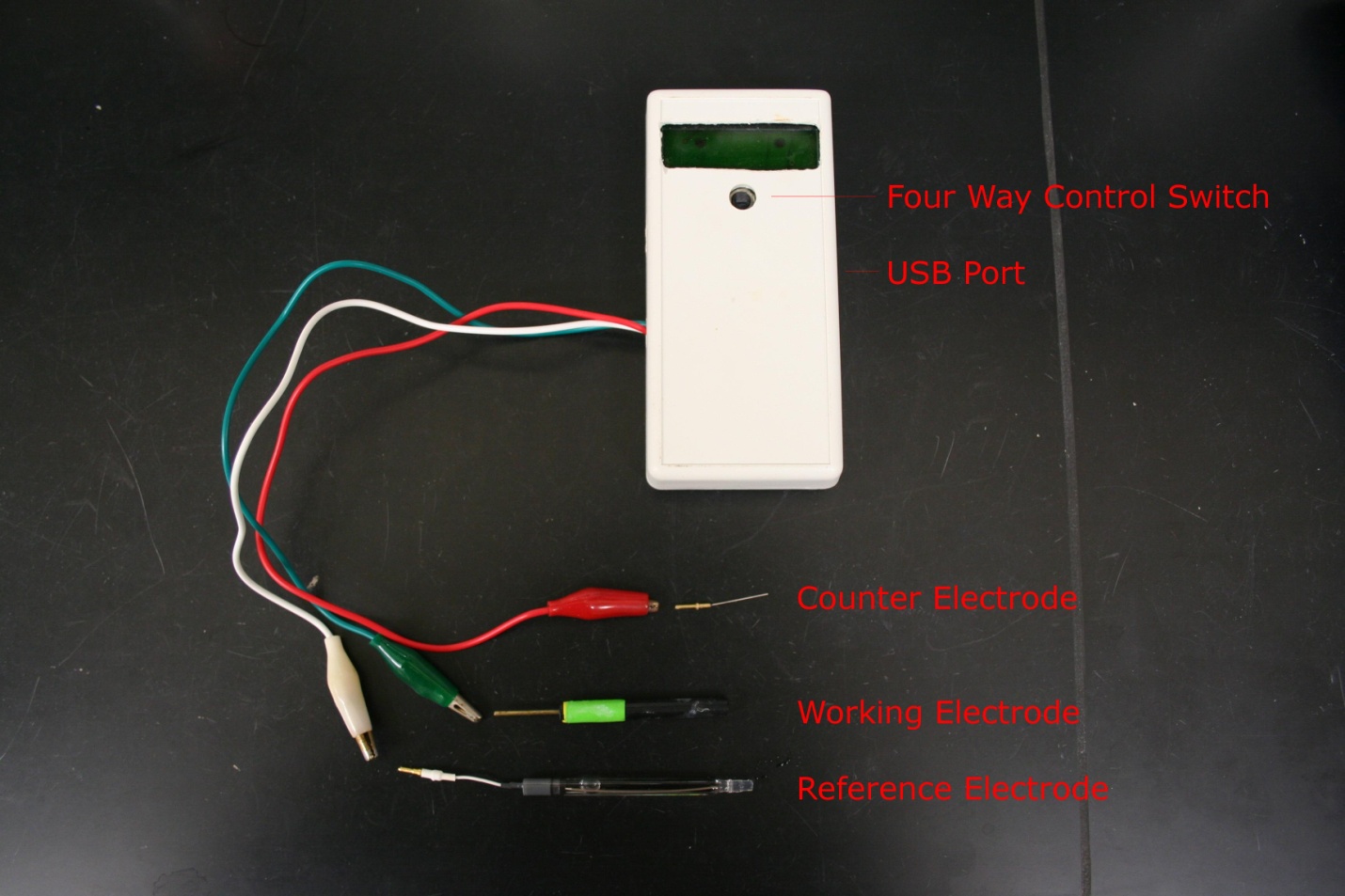


Figure S7 – Key features of the device. The red wire should be attached to a counter electrode. The green wire can be attached to a working electrode. The white wire can be attached to a reference. The device is controlled with a four way switch, and there is a USB port on the side, which supplies power and an interface with any netbook, laptop, or desktop computer.

Table S1 -- Cheapstat Version 2.0

Complete Parts List


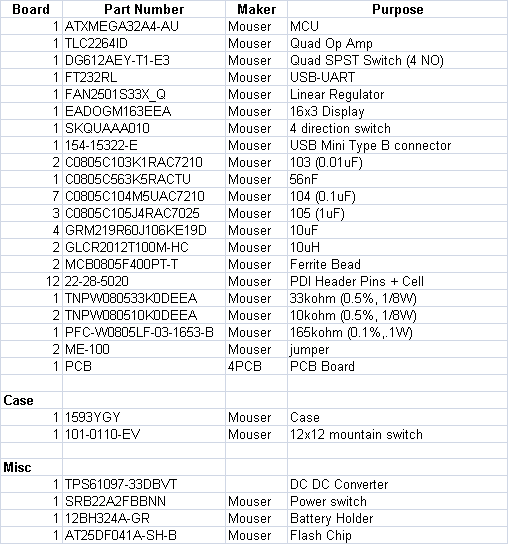


**Key Features of the CheapStat**

Square Wave Voltammetry

Alternating Current Voltammetry

Linear Sweep Voltammetry

Constant Potential Mode

Frequency Range: 1-1000Hz

Voltage Range: -990 to 990mV

Current Range: ~100nA to ~10µA

**Note for Early Adopters**

For information demonstrating proper use of the CheapStat, as well as updates to this design, circuit board layout, and source code, can be found on the website of Professor Kevin Plaxco, where it is available free of charge.

http://www.plaxcolab.com/cheapstat

or

http://www.chem.ucsb.edu/~kwp/cheapstat/

Feel free to email us with questions. We’re friendly!

We welcome design suggestions and code.
